# Highly selective polymer electrolyte membranes consisting of poly(2-ethyl-2-oxazoline) and Cu(NO_3_)_2_ for SF_6_ separation

**DOI:** 10.1038/srep20430

**Published:** 2016-02-10

**Authors:** Woong Gi Lee, Sang Wook Kang

**Affiliations:** 1 Department of Chemistry, Sangmyung University, Seoul 110-743, Republic of Korea

## Abstract

Polymer electrolyte membranes consisting of Cu(NO_3_)_2_ and poly(2-ethyl-2-oxazoline) (POZ) were prepared for SF_6_/N_2_ separation. It was anticipated that repulsive forces would be operative between the negative charge of water and the F atoms of SF_6_ when Cu(NO_3_)_2_ in the composite was solvated by water, and that the barrier effect of Cu^2+^ ions would be activated. In fact, Cu(NO_3_)_2_ solvated by water in the POZ membrane was observed to have more higher-order ionic aggregates than free ions or ion pairs, as confirmed by FT-Raman spectroscopy. Thus, when Cu(NO_3_)_2_ solvated by water was incorporated into the POZ matrix, the N_2_/SF_6_ selectivity increased to 28.0 with a N_2_ permeance of 11.2 GPU at a POZ/Cu(NO_3_)_2_ mole ratio of 1:0.7. The coordinative interaction of Cu(NO_3_)_2_ with the carbonyl group in POZ was confirmed by FT-IR spectroscopy and TGA, and the film thickness of the membrane was determined from SEM analysis.

Separation of gas mixtures is very important in industrial fields^1^. Sulfur hexafluoride (SF_6_) gas is widely used in a variety of industrial processes, but it is one of the most potent greenhouse gases^2^. SF_6_ separation is very important given that its green house gas effect is 23900 times higher than that of CO_2_ gas, and SF_6_ is also a very stable gas with an atmospheric lifetime of 3200 years^3^,^4^. To resolve these issues, techniques for SF_6_ separation and decomposition have recently gained interest^5^.

Many studies have focused on developing technologies for separation of SF_6_ and its derivatives, some of which have been partially commercialized. As an example of an SF_6_ separation technique, a hollow fiber membrane was fabricated using a dry-wet phase inversion method. Separation of a N_2_/SF_6_ binary mixture using the hollow fiber membrane was investigated^6^. Furthermore, the plasma, electrical discharge, and spark methods are quite efficient methods for decomposition of SF_6_^7^,^8^. In another study, different porous materials were evaluated as absorbents for SF_6_. Stainless steel slag (SSS), which is rich in metal and silicon oxide, was reported to be an effective SF_6_ decomposition agent^9^. Multi-walled carbon nanotubes (MWNTs) have also been evaluated for adsorption of SF_6_. Furthermore, zeolites, silicalite, and pillared clays were studied for adsorption of C1 to C4 alkanes and SF_6_ gas^10^,^11^,^12^,^13^. Metal organic frameworks (MOFs) such as DUT-9, Cu-BTC, and MIL-101 and porous coordination polymers (PCPs) have been used to separate SF_6_, CO, CO_2_, and CH_4_^14^,^15^,^16^,^17^,^18^.

Recently, gas separation has been widely studied using the electrolyte membrane. For example, the greenhouse gases CO_2_ and CH_4_ were separated from CO_2_/N_2_ and CO_2_/CH_4_ mixtures by using a polymer electrolyte. The polymeric gas separation membrane exhibited good performance. In one study, CO_2_ was separated from a CO_2_/N_2_ mixture by using an electrolyte membrane with potassium fluoride (KF) in poly(vinylpyrrolidone) (PVP) solution. The potassium ion of KF was coordinated with the amide group of the PVP polymer to induce crosslinking. The interaction between the K^+^ and CO_2_ molecules facilitated CO_2_ transport, resulting in enhancement of the CO_2_ separation performance. As a result, the CO_2_ permeance was 28.0 GPU and the selectivity (CO_2_/N_2_) was 4.1^19^.

In this study, we report the fabrication of an electrolyte membrane by simply adding Cu(NO_3_)_2_ to poly(2-ethyl-2-oxazoline) (POZ). The N_2_ and SF_6_ gas permeance of the electrolyte membrane is evaluated as shown in Fig. 1, demonstrating that the electrolyte membrane containing Cu(NO_3_)_2_ exhibits excellent separation performance. Cu(NO_3_)_2_ incorporated into the POZ membrane was found to have more higher-order ionic aggregates than free ions or ion pairs, as confirmed by FT-Raman analysis.

## Results and Discussion

### FT-IR

FT-IR measurements were performed on a Varian FTS3100 spectrometer; 64–200 scans were averaged at a resolution of 4 cm^−1^. Thermogravimetric analysis (TGA) was performed with Mettler Toledo TGA devices at a heating rate of 10 °C/min. FT-Raman measurements were performed for Cu(NO_3_)_2_ and POZ/Cu(NO_3_)_2_ solutions at room temperature using a Horiba Jobin Yvon LabRam Aramis with a diode laser beam at an excitation wavelength of 785 nm.

The aggregates of copper nitrate were dispersed in the polymer chain. The ion aggregates were solvated by the partial positive charge of water. Therefore, the partial negative charge of water was localized on the exterior of the ion aggregates. Non-polar N_2_ gas was transported through the polymer membrane by Fickian transport, but SF_6_ gas having a high quadruple moment experienced higher repulsion with the partial negative charge of water. Furthermore, the SF_6_ molecules could be blocked by activated Cu cations in the polymer chains.

**Figure 2:**
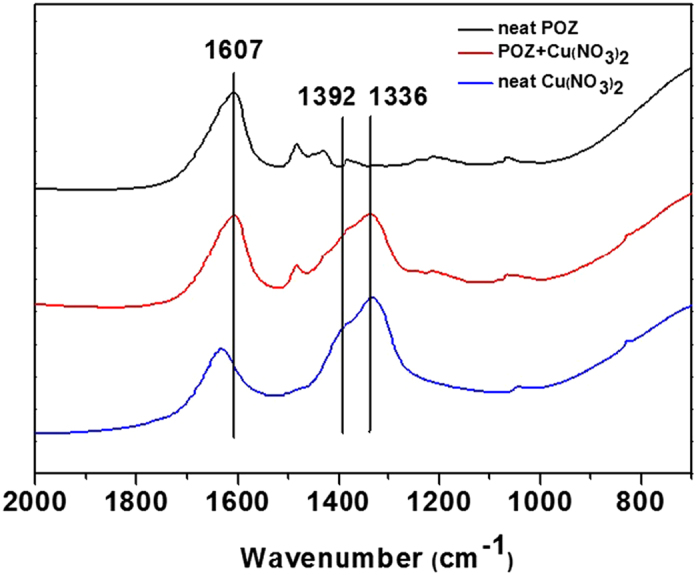
FT-IR spectra of neat POZ and POZ/Cu(NO_3_)_2_ electrolyte.

To confirm the formation of the polymer electrolytes consisting of POZ and Cu(NO_3_)_2_, the interaction between copper ions and the carbonyl groups in the amide of the polymer was investigated using FT-IR spectroscopy. Figure 2 shows that the spectrum of neat POZ has an intense free C=O peak at 1607 cm^−1^. When Cu(NO_3_)_2_ was incorporated into POZ, the shift of the free C=O peak at 1607 cm^−1^ was negligible. Thus, the copper ion appeared not to interact with the carbonyl group. Furthermore, it was known that the stretching frequency of free NO_3_^−^ occurs at 1336 cm^−1^. When Cu(NO_3_)_2_ was incorporated into POZ, the intensity of the peak at 1392 cm^−1^, which is the aggregated NO_3_^−^, diminished and the peak of free NO_3_^−^ at 1336 cm^−1^ became dominant. This observation indicated that the ionic bonding of nitrate ions with the copper ions became weakened by interaction with the hydrogen bonds formed with water, resulting in the observed decrease of the stretching frequency.

### Thermal analysis

**Figure 3:**
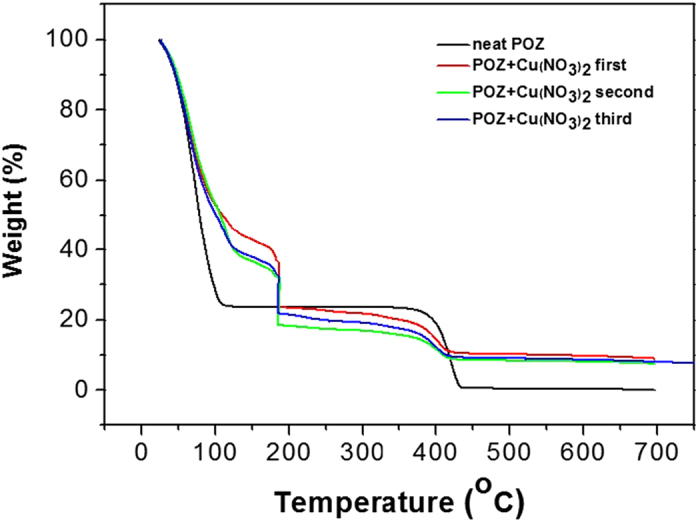
TGA analyses of neat POZ and POZ/Cu(NO_3_)_2_ (1:0.7 mol ratio).

Thermogravimetric analysis (TGA) was carried out to confirm the thermal stability of the polymer electrolyte using a Universal V4.5A (TA Instruments) apparatus. Figure 3 shows the TGA data for neat POZ and POZ/Cu(NO_3_)_2_ (1:0.7 mol ratio), demonstrating 78% weight loss for neat POZ between 0 °C and 100 °C, attributed to evaporation of water. On the other hand, when copper nitrate was incorporated into the POZ polymer, water evaporation progressed at a higher temperature of above 100 °C, possibly due to solvation of copper nitrate by water. Therefore, the increase in the decomposition temperature was attributed to the strong interaction of copper with water. The boiling point of copper nitrate is known to be 170 °C. Thus, the loss of about 20% weight at about 170 °C was thought to be due to copper nitrate. Also, weight loss was apparent for all samples at around 400 °C due to degradation of the POZ polymer. It is deduced from this result that copper nitrate did not interact with the POZ polymer. Copper nitrate was solvated by water and thus the decomposition temperature of copper nitrate and water increased.

### FT-Raman spectroscopy

**Figure 4:**
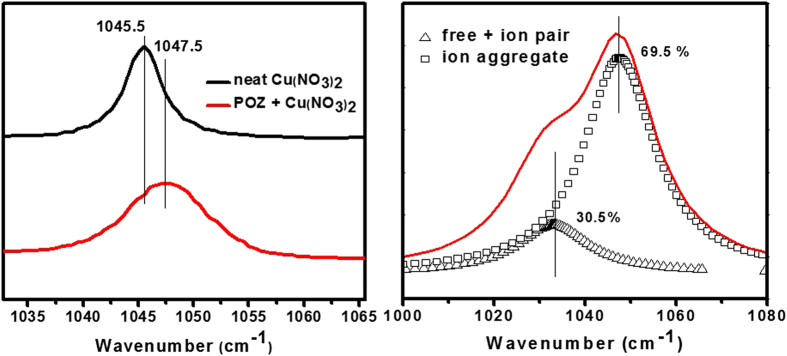
FT-Raman spectra of (**a**) neat Cu(NO_3_)_2_ and POZ/Cu(NO_3_)_2_ (1:0.7 mol ratio) and (**b**) the deconvoluted spectra for POZ/Cu(NO_3_)_2_.

To investigate the ionic constituents (i.e. free ions, ion pairs, and higher-order aggregates) of NO_3_^−^ in neat Cu(NO_3_)_2_ and POZ with Cu(NO_3_)_2_, FT-Raman spectra were acquired as shown in Fig. 4. The ionic constituents of NO_3_^−^ are known to present stretching bands at 1034, 1040, and 1045 cm^−1^, assigned to free ions, ion pairs, and ion aggregates, respectively^20^. When copper nitrate was incorporated into the POZ polymer, the wavenumber of the NO_3_^−^ species increased negligibly from 1045.5 to 1047.5 cm^−1^ as shown in Fig. 4(a), indicating that Cu(NO_3_)_2_ incorporated into POZ existed mostly as ion aggregates. In the case of Cu(NO_3_)_2_ incorporated into POZ, the relative percentage of free ions, ion pairs, and aggregates is shown in Fig. 4(b). New peaks were observed at 1029 and 1034 cm^−1^. These peaks were attributed to free ions and ion pairs. The peak areas for each configuration were measured to be 14.829, 15.664, and 69.501% as shown Fig. 4(b). Based on these results, Cu(NO_3_)_2_ incorporated into the POZ polymer formed more higher-order ion aggregates than free ions or ion pairs and the ion aggregates were dispersed in the membrane chain.

### Structural morphology

**Figure 5:**
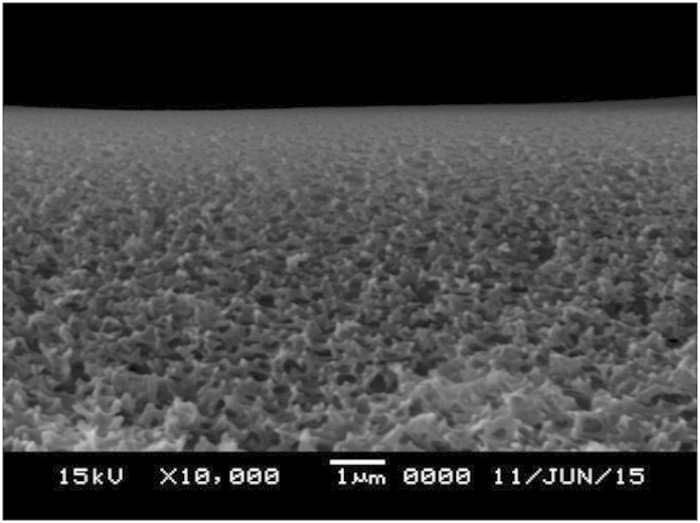
SEM image of POZ/Cu(NO_3_)_2_ (1:0.7 mol ratio) electrolyte polymer membrane.

Scanning electron microscopy (SEM) was used to investigate the thickness of the polymer electrolyte on the polysulfone macroporous membrane supports, as shown in Fig. 5. When the polymer electrolyte solution was coated onto the supports, the thickness of the selective layer was about 1 μm.

### Separation performance

**Figure 6:**
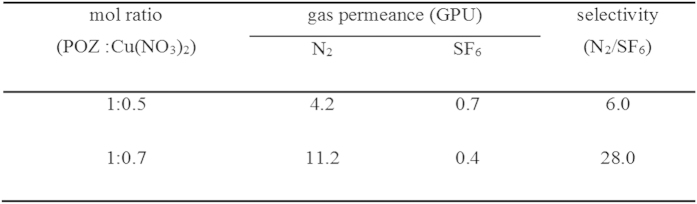
Gas permeance and selectivity of polymer electrolyte at different compositions.

**Figure 7:**
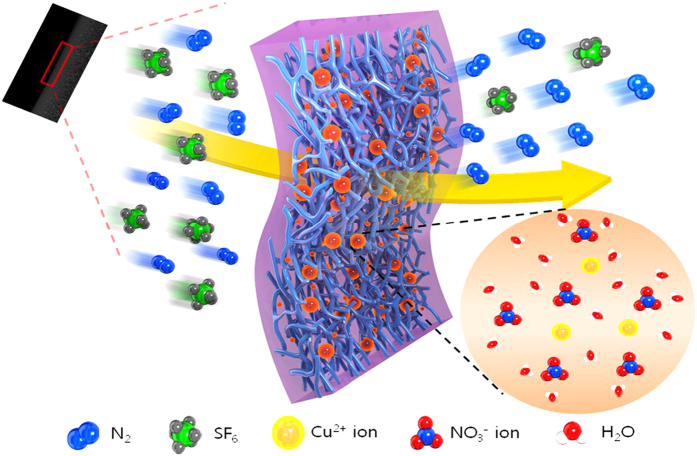
Polymer electrolyte membrane for SF_6_ separation.

After the electrolyte membrane was coated with the control coater, the membrane was applied as the SF_6_ separation membrane. Figure 6 showed the single gas permeance and selectivity of N_2_/SF_6_ through the copper nitrate ion aggregate membrane. The N_2_ gas permeance at each mol ratio (1:0.5 to 1:0.7) was about 4.2 and 11.2 GPU while the SF_6_ gas permeance was about 0.7 and 0.4 GPU. 1:0.7 mol ratio of POZ/Cu(NO_3_)_2_ gave rise to the highest N_2_ gas permeance with the lowest SF_6_ gas permeance. In addition, after 2 d, the SF_6_ gas permeance decreased to about 0.2 GPU. Therefore, the selectivity of the POZ/Cu(NO_3_)_2_ (1:0.7 mol ratio) membranes was the best at 28.0. Thus, because Cu(NO_3_)_2_ was solvated by water, the repulsive force between the negative charge of water and the F atoms of SF_6_ increased, and the barrier effect of activated Cu^2+^ ions was also enhanced as shown in Fig. 7.

This study presented a simple method for the preparation of an electrolyte membrane containing Cu(NO_3_)_2_ aggregates as a barrier material. The membrane was simply fabricated by adding Cu(NO_3_)_2_ to the POZ solution followed by preparation of the composite by using a control coater. The N_2_ permeance achieved with a 1:0.5 mol ratio of POZ/Cu(NO_3_)_2_ was 4.2 GPU, which increased to 11.204 GPU in the case of a 1:0.7 mol ratio of POZ/Cu(NO_3_)_2_. On the other hand, the SF_6_ permeance decreased at a 1:0.7 mol ratio of POZ/Cu(NO_3_)_2_. Therefore, the highest selectivity of 28.0 was achieved with a 1:0.7 mol ratio of POZ/Cu(NO_3_)_2_.

## Methods

### Materials

Poly(2-ethyl-2-oxazoline) (POZ) and copper nitrate hydrate (Cu(NO_3_)_2_·xH_2_O) were purchased from Sigma-Aldrich and used without further purification.

### Fabrication process

A polymer solution was prepared by dissolving POZ in water (20 wt%). To prepare the polymer electrolyte solution, the amount of Cu(NO_3_)_2_·xH_2_O added to the polymer matrix was determined according to the mole ratio of the copper ion to the monomeric unit of POZ. This solution was stirred for one day at room temperature. This polymer electrolyte solution was coated onto polysulfone macroporous membrane supports (Toray Inc., Japan) using an RK Control Coater (Model 101, Control Coater RK Print-Coat Instruments Ltd., UK). To contain the water moistly, the polymer electrolyte membranes were dried for 10 minutes at room temperature. The gas flow rates were measured using a bubble flow meter. The unit of gas permeance is GPU, where 1 GPU = 1 × 10^−6^ cm^3^ (STP)/(cm^2^·s·cmHg).

## Characterization

FT-IR measurements were performed on a Varian FTS3100 spectrometer; 64–200 scans were averaged at a resolution of 4 cm^−1^. Thermogravimetric analysis (TGA) was performed with Mettler Toledo TGA devices at a heating rate of 10 °C/min. FT-Raman measurements were performed for Cu(NO_3_)_2_ and POZ/Cu(NO_3_)_2_ solutions at room temperature using a Horiba Jobin Yvon LabRam Aramis with a diode laser beam at an excitation wavelength of 785 nm.

## Additional Information

**How to cite this article**: Lee, W. G. and Kang, S. W. Highly selective polymer electrolyte membranes consisting of poly(2-ethyl-2-oxazoline) and Cu(NO_3_)_2_ for SF_6_ separation. *Sci. Rep.* **6**, 20430; doi: 10.1038/srep20430 (2016).

## Figures and Tables

**Figure 1:**
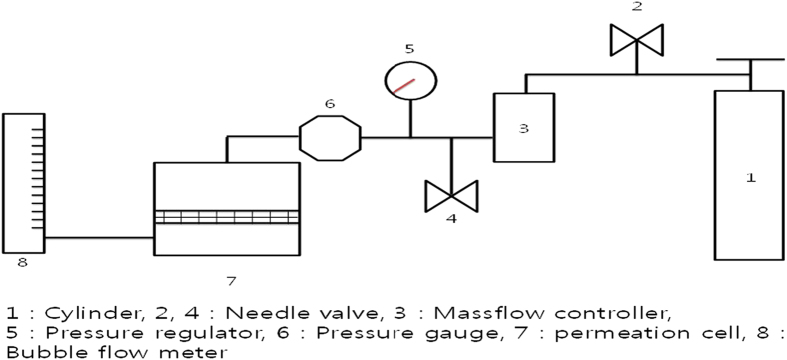
Experimental set-up for the gas permeation test.
